# Bridging the Gap: Investigating the Link between Inflammasomes and Postoperative Cognitive Dysfunction

**DOI:** 10.14336/AD.2023.0501

**Published:** 2023-12-01

**Authors:** Siyu Zhang, Cuiying Liu, Jintao Sun, Yang Li, Jian Lu, Xiaoxing Xiong, Li Hu, Heng Zhao, Hongmei Zhou

**Affiliations:** ^1^Anesthesiology Department, Zhejiang Chinese Medical University, Hangzhou, China.; ^2^Anesthesiology Department, The Second Hospital of Jiaxing, The Second Affiliated Hospital of Jiaxing University, Jiaxing Key Laboratory of Basic Research and Clinical Transformation of Perioperative Precision Anesthesia, Jiaxing, China.; ^3^School of Nursing, Capital Medical University, Beijing, China.; ^4^Beijing Institute of Brain Disorders, Laboratory of Brain Disorders, Ministry of Science and Technology, Joint Innovation Center for Brain Disorders, Capital Medical University, Beijing, China.; ^5^Department of Neurosurgery, Renmin Hospital of Wuhan University, Wuhan, China

**Keywords:** POCD, inflammasomes, NLRP3, cognitive disorder, neuroinflammation, surgery

## Abstract

Postoperative cognitive dysfunction (POCD) is a cluster of cognitive problems that may arise after surgery. POCD symptoms include memory loss, focus inattention, and communication difficulties. Inflammasomes, intracellular multiprotein complexes that control inflammation, may have a significant role in the development of POCD. It has been postulated that the NLRP3 inflammasome promotes cognitive impairment by triggering the inflammatory response in the brain. Nevertheless, there are many gaps in the current literature to understand the underlying pathophysiological mechanisms and develop future therapy. This review article underlines the limits of our current knowledge about the NLRP3 (NOD-, LRR- and pyrin domain-containing protein 3) inflammasome and POCD. We first discuss inflammasomes and their types, structures, and functions, then summarize recent evidence of the NLRP3 inflammasome's involvement in POCD. Next, we propose a hypothesis that suggests the involvement of inflammasomes in multiple organs, including local surgical sites, blood circulation, and other peripheral organs, leading to systemic inflammation and subsequent neuronal dysfunction in the brain, resulting in POCD. Research directions are then discussed, including analyses of inflammasomes in more clinical POCD animal models and clinical trials, studies of inflammasome types that are involved in POCD, and investigations into whether inflammasomes occur at the surgical site, in circulating blood, and in peripheral organs. Finally, we discuss the potential benefits of using new technologies and approaches to study inflammasomes in POCD. A thorough investigation of inflammasomes in POCD might substantially affect clinical practice.

## 1. Introduction

Postoperative cognitive dysfunction (POCD) is a frequent clinical disorder that affects many patients following surgery, especially those who are elderly or have preoperative dementia [1-7]. It manifests as a loss in cognitive capacity, including memory, attention, and executive function, and can last for weeks to months following surgery [6-9]. With over 310 million surgical procedures performed annually on a global scale [10, 11], and the number of surgical procedures rising in rapidly aging populations such as in China [12, 13], POCD is a primary concern for patient outcomes. In addition, it is one of the many problems connected with operation trauma, which can substantially influence life quality and daily independent activities [14].

While the precise pathophysiology of POCD is not completely known, there is growing evidence that neuroinflammation plays a significant role [5, 15, 16]. Inflammation is a fundamental immunological reaction to tissue damage, infection, or injury. It is characterized by the activation of immune cells, including macrophages and neutrophils, which release pro-inflammatory cytokines like TNF-α, IL-1β, and IL-6 [17-21]. Inflammation is necessary for tissue healing and pathogen elimination, but it can be harmful if it is dysregulated or prolonged [22-24]. For instance, surgical trauma is an acute stress response to sterile injury induced by surgery; this causes inflammation that can disrupt the sympathetic nervous system, neuroendocrine system, and hypothalamus-pituitary-adrenal axis, leading to systemic inflammation and immune system dysfunction [25-27]. In addition, it is well known that inflammation is regulated by inflammasomes, which are multiprotein complexes involved in the control of innate immunity and have been linked to the etiology of numerous human disorders, including POCD [28-35]. Thus, the potential role of inflammasomes in POCD has received much attention.

Recent studies have established a strong link between NLRP3 (NOD-, LRR- and pyrin domain-containing protein 3) inflammasome-mediated neuroinflammation and POCD [29, 36-39]. The NLRP3 inflammasome is an intracellular complex of proteins that is part of the inflammatory pathway of the immune system [18, 40-43]. It comprises the sensor of an NLR, the adaptor of ASC, and the effector of an inflammatory caspase-1 processing inactive cytokine preforms. The NLRP3 inflammasome has been found to contribute to the progression of POCD directly and is closely linked with high-risk factors, such as postoperative infection, pain, and preoperative mental disorders [36, 37, 39, 44-46].

Although the relationship between the NLRP3 inflammasome and POCD has been extensively studied, substantial gaps in our knowledge still need to be addressed. For instance, there are several types of inflammasomes, including NLRP1, NLRP3, and NLRC4, among others, but in the context of POCD, only the NLRP3 inflammasome has been extensively examined [29, 36, 44]. In addition, surgical trauma results in inflammation and produces pro-inflammatory cytokines at the surgical site; thus, it is very likely that inflammasomes may also involve in the surgical site; however, the NLRP3 inflammasome was only studied in the brain in the context of POCD. Furthermore, previous studies have mainly used animal models. While it has provided crucial insights into the role of inflammasomes in POCD [47-49], few clinical investigations have been conducted to test the potential role of inflammasomes in POCD. Hence, there is a need for additional research into the association between POCD and inflammasomes, especially in understudied areas such as peripheral organs and clinical investigations.

This review seeks to summarize the current understanding of inflammasomes' involvement in POCD and discuss prospective routes for future research. We will first provide an overview of inflammasomes of various types and their structure and activities and will then re-examine evidence that inflammasomes in POCD. After that, we will form our new hypothesis and discuss future research directions. By emphasizing the importance of investigating other inflammasomes, examining their roles in peripheral organs, and conducting clinical studies, we aim to fill knowledge gaps in the literature and identify new intervention targets. This review will pave the way for developing new experiments to clarify POCD pathophysiological mechanisms and ultimately contribute to our understanding of the pathogenesis of POCD.

## 2. An overview of inflammasome classification, constituents, and activation

**Figure 1.**
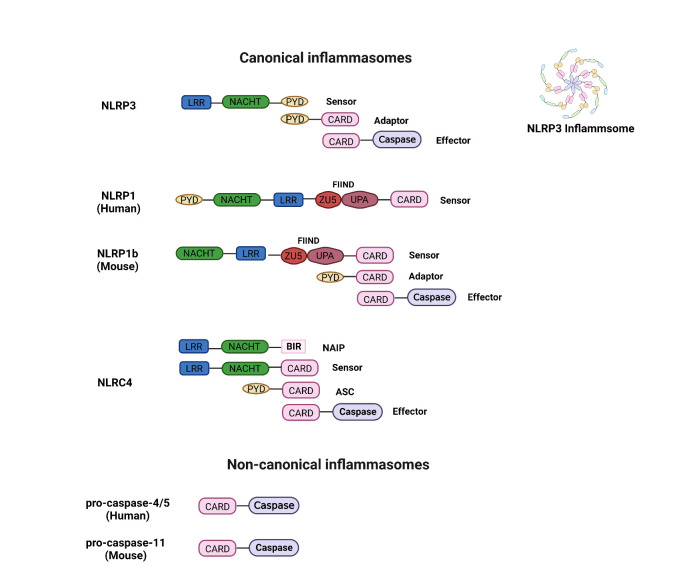
**The structure and components of inflammasomes**. Inflammasomes can be classified as canonical and noncanonical inflammasomes. The former contains a sensor, adaptor, and effector, while the latter does not have adaptors. The structures of NLRP3, NLRP1 (human) and NLRP1b (mouse), and NLRC4 are presented for the canonical inflammasomes. For example, the NLRP3 inflammasome, as a canonical inflammasome, contains NLRP3, ASC, and pro-caspase-1. Furthermore, the NLRP3 protein comprises a C-terminal LRR domain, a central nucleotide-binding and oligomerization domain (NACHT), and an N-terminal PYD. The ASC protein contains PYD and CARD domains. Pro-caspase-1 is formed by a CARD domain and a catalytic domain known as the caspase domain. Upon activation in the LRR domain, the amino-terminal PYD interacts with the PYD domain of ASC and then recruits pro-caspase-1 monomer through CARD-CARD interaction, finally forming the NLRP3 inflammasome. In addition, the structures of pro-caspase-4/5 (human) and pro-caspase-11 (mouse) are shown for the noncanonical inflammasomes.

Inflammasomes are cytosolic multiprotein complexes, which can be classified as canonical inflammasomes and noncanonical inflammasomes [40, 50, 51] (Fig. 1). The canonical inflammasomes are often composed of three components: a sensor that is typically a cytoplasmic pattern-recognition receptor (PRR), an effector that is pro-caspase-1, and an adapter that binds the sensor and effector together [50, 52, 53] (Fig. 1). Their sensors, such as NLRP3, NLRC4, and NLRP1, define and name the subtypes of inflammasomes [54, 55]. A sensor PRR in the inflammasome detects various stimuli in the tissue, an adapter aids in assembling inflammasomes, and a caspase effector produces inflammatory factors to induce inflammation [56]. Nevertheless, an adaptor is not required for noncanonical inflammasomes, in which the caspase component can serve as both sensor and effector (Fig. 1) [57, 58].

The vast majority of inflammasome sensors are members of the nucleotide-binding domain and leucine-rich repeat (NLR) family, including NLRP3, NLRC4, NLRP1 (human), or NLRP1b (mouse), which consists of a nucleotide-binding domain (NBD) and a leucine-rich repeat (LRR) receptor [17, 40, 52, 59-61]. The NBD includes the nucleotide-binding and oligomerization (NACHT) or nucleotide-binding oligomerization (NOD) domain (Fig. 1). The NACHT domain is named after the proteins in which it was initially identified: neuronal apoptosis inhibitory protein (NAIP), CIITA, HET-E, and TP1 [62]. The NOD is used interchangeably with NACHT [63]. The NACHT/NOD domain in NLR proteins is responsible for nucleotide binding and oligomerization, which are critical steps for inflammasome assembly and activation. When NLR proteins sense specific pathogen-associated molecular patterns (PAMPs) or damage-associated molecular patterns (DAMPs), the NACHT/NOD domain binds to ATP, which leads to a conformational change in the protein. This change enables the protein to oligomerize and form the core structure of the inflammasome [40, 60, 64]. The activated inflammasome then facilitates the cleavage and release of pro-inflammatory cytokines, such as IL-1β and IL-18, leading to an inflammatory response.

Thus, the NLR family consists of the subgroups NLRP and NLRC. NLRPs and NLRCs differ at their N-termini, with NLRPs' sensor containing a pyrin domain (PYD) and NLRCs' sensor comprising a caspase recruitment domain (CARD) [40, 52, 60, 64]. Nevertheless, the ability of the NLR sensors NLRP1, NLRP3, and NLRC4 to form inflammasomes is functionally related. Other non-NLR sensors, such as absent-in-melanoma-2 (AIM2) and pyrin, can also form inflammasomes [65-69].

Adaptors include apoptosis-associated speck-like protein (ASC) and NIMA-related protein kinase 7 (NEK7) [54, 70]. ASC contains one CARD and one PYD, which facilitate interaction between the inflammasome sensor and pro-caspase-1. ASC is recruited by inflammasome sensors that lack a CARD domain, such as NLRP3 and AIM2, but is not required for the formation of NLRP1 and NLRC4 [71, 72]. When inflammasome activation is initiated, the proteins of ASC form a single large, micrometer-sized, speck-like structure in the cell, allowing caspase-1 to concentrate in this site [73, 74] (Fig. 1). Another adapter, NEK7, is a serine-threonine kinase, exerts a scaffolding function by bridging adjacent NLRP3 molecules through binding to their LRR domain, which is essential for NLRP3 activation [44, 70].

**Figure 2.**
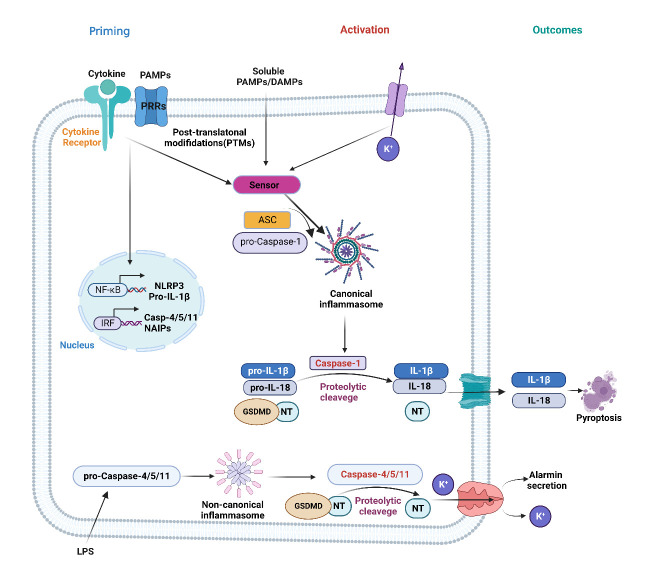
**The priming and activation steps of inflammasome activation**. In the priming stage, cytokines and PAMPS trigger cytokine receptors or PRRs, leading to NF-κB translocation into the nucleus, initiating the transcription of NLRP3 and the immature forms of inflammatory cytokines. In signal two activations, multiple endogenous and exogenous stimuli induce sensors such as NLRP3 to recruit ASC and oligomerize NLRP3 through homotypic interaction between the PYD regions of the two proteins. Pro-caspase-1 is then recruited to oligomeric ASC through homotypic interaction between the CARD regions, ultimately promoting NLRP3 inflammasome formation-the dimerization of pro-caspase-1 triggers autolysis, producing two active caspase-1 enzymes that mature pro-IL-1β and pro-IL-18. In addition to the canonical inflammasome activation pathway, a noncanonical path can be triggered by stimuli such as bacterial LPS or cytosolic lipids. In this pathway, LPS recognition leads to caspase-4/5/11 activation, which cleaves GSDMD and releases GSDMD-NT, forming oligomers in the plasma membrane that allow the release of pro-inflammatory cytokines, K^+^ efflux, and the secretion of alarmins, such as HMGB1. DAMPs: danger-associated molecular patterns; PAMPs: pathogen-associated molecular patterns; NF-κB: Nuclear factor κB.

Canonical and noncanonical inflammasomes can be distinguished by whether caspase-1 is involved in their formation [54]. The sensors NLRP1, NLRP3, and NLRC4 recruit caspase-1 to form canonical inflammasomes, with or without the ASC adaptor, whereas human caspase-4/5 or murine caspase-11 serves as both sensor and effector in noncanonical inflammasomes. An adaptor is not required for noncanonical inflammasomes [75, 76].

All inflammasomes are activated by a two-step activation model, priming and activation, despite the diversity of inflammasomes and the uniqueness of their sensors, which are associated with different platform mechanisms [40] (Fig. 2). Upon priming by various stimuli, proteins required for inflammasome assembly (such as NLRP3) and downstream signaling (such as pro-IL-1β) will be upregulated at transcriptional levels. Primed sensors are auto-repressed but in a signal-sensitive state that a variety of PAMPs or DAMPs can trigger. During the second step's activation, the primed sensor undergoes post-translational modifications (PTMs), resulting in a derepressed conformational change that initiates sensor oligomerization and inflammasome platform assembly [40].

Best studied is the two-step model for the NLRP3 inflammasome [18, 42, 51, 54, 77]. Toll-like receptors (TLRs), NOD2, and the receptor for advanced glycation end products can prime the NLRP3 sensor [42]. Additionally, it can be primed by anaphylatoxin C3a and C5a receptors, cytokine IL-1, and TNF receptors, and excessive reactive oxygen species (ROS) production [78-80]. These priming processes result in the upregulation of NLRP3, pro-IL-1, inflammasome substrate IL-18, and Gasdermin D(GSDMD) [61, 81]. Furthermore, priming enables NLRP3 to subsequent activation by many agents and stress conditions, including bacterial toxins such as nigericin and the membrane attack complex assembled upon the activation of the complement system, extracellular ATP, asbestos, silica, cholesterol crystals, β-amyloid, and liposomes [81]. A common mechanism upstream of NLRP3 activation control is observed: a reduction in cytosolic K^+^ levels when macrophages are exposed to extracellular ATP, pore-forming toxins, and particles [82]. Upon activation, NLRP3 undergoes post-translational modifications, initiating the formation of inflammasomes with ASC and pro-caspase-1.

Caspase-1, a cysteine protease, is initially generated as an inactive pro-caspase-1 zymogen comprised of an N-terminal CARD domain and two catalytic subunits, p20 and p10 [29, 75, 83, 84]. Following recruitment to canonical inflammasomes via its CARD domain, pro-caspase-1 undergoes autolytic activation and releases p20 and p10, the forms of active caspase-1 [83]. Activated caspase-1 cleaves pro-IL-1β and pro-IL-18 cytokines and Gasdermin D into their physiologically active forms, IL-1β and IL-18, and N-terminal domain of GSDMD (GSDMD-NT), respectively [83, 85]. While IL-1β and IL-18 are essential for the ensuing inflammatory response, the released GSDMD-NT translocates to the plasma membrane to generate membrane holes, permitting mature IL-1β and IL-18 secretion into extracellular space, leading to pyroptotic cell death. In addition, the gasdermin pore also allows the release of alarmins, including IL-1α [86] and high mobility group box-1 protein (HMGB1) [87], which further exacerbates inflammatory responses. Although other inflammasomes, such as NLRC4 inflammasome and caspse-11 and caspase-4/5 non-canonical inflammasomes have different platforms for their inflammasome assembly, they all produce similar inflammatory products such as IL-1β and IL-18, and result in pyroptosis through the similar steps of the two-step activation model [55, 70, 88, 89].

## 3. Currently available evidence on the role of inflammasomes, mainly the NLRP3 inflammasome, in POCD

Inflammasomes, particularly the NLRP3 inflammasome, have been implicated in the pathophysiology of POCD, according to research in animal models [38, 39, 42, 90, 91]. In addition, surgery trauma activates the immune system in animal models of POCD, which produces pro-inflammatory cytokines that may cause neuro-inflammation and cognitive deficits [29, 38, 39, 92]. According to these results, several inflammasomes may contribute to the onset of POCD, and targeting these pathways may be therapeutically effective.

Activation of the NLRP3 inflammasome has been found to significantly impact the development of cognitive difficulties in individuals with POCD. Researchers have discovered that activating the NLRP3 inflammasome may produce pro-inflammatory cytokines, which can exacerbate neuroinflammation and neuronal injury [29, 38, 39]. In addition, the NLRP3 inflammasome has been demonstrated to activate microglia, immune cells in the central nervous system, and to trigger the production of neurotoxic chemicals [39]. Only rodent models have been used to determine the function of the NLRP3 inflammasome in POCD. Most of these research studies employed anesthetic alone to induce or simulate POCD. Thus, we will examine the involvement of the NLRP3 inflammasome in POCD depending on how the animal models were constructed.

## 3.1. Function of NLRP3 inflammasome in POCD produced by anesthesia alone in animal models

### 3.1.1 Isoflurane-induced POCD

Numerous studies have addressed the involvement of NLRP3 inflammasome in isoflurane-induced POCD in mouse models. One of the most comprehensive studies was conducted by Wang et al., who used a POCD model induced by isoflurane without surgery in young and aged mice [29]. They found that the NLRP3 protein increased in the hippocampus of aged mice but not in young mice, suggesting that age is a critical factor for POCD, consistent with clinical observations. Additionally, caspase-1 levels and cleaved IL-1β and IL-18 were elevated in aged mice by isoflurane treatment, and these alterations were reversed by pretreatment with the caspase-1 inhibitor Ac-YVAD-cmk. Their data indicate that the activation of the NLRP3 inflammasome may contribute to isoflurane-induced hippocampus inflammation. To corroborate this, the authors conducted in vitro investigations utilizing BV-2 cells and primary microglial cell culture, in which LPS was utilized to stimulate the NLRP3 inflammasome. They observed that LPS generated a dose-dependent rise in NLRP3, but not in IL-1β mRNA, suggesting that particular doses of LPS primed microglial cells. Additionally, NLRP3 priming was essential for isoflurane-induced NLRP3 inflammasome activation, because following LPS priming, isoflurane treatment generated a substantial increase in IL-1β and IL-18 production; however, isoflurane alone could not. Finally, in vitro NLRP3 knockdown attenuated the isoflurane-induced NLRP3 inflammasome activation [29].

More direct evidence of the NLPR3 inflammasome in isoflurane-induced POCD was provided by Zhang et al., who employed lentivirus-specific short hairpin RNA to reduce NLRP3 expression in aged rats [93]. In this study, NLRP3 was designated as PYRIN-containing Apaf1-like protein 1 (PYPAF1). The scientists reported that PYPAF1 knockdown alleviated cognitive impairment. Furthermore, isoflurane exposure enhanced PYPAF1 (i.e., NLRP3) and ASC expression, but silencing PYPAF1 mitigated this impact. Additionally, isoflurane administration increased the activation of microglia and caspase-1 and IL-1β and IL-18 production, all of which were blocked by PYPAF1 silencing. Hence, the NLRP3 inflammasome likely contributes to POCD.

Two further investigations offered indirect evidence about the participation of the NLRP3 inflammasome in isoflurane-induced POCD [37, 46]. First, Que et al. examined the involvement of dual-specificity phosphatase 14 (DUSP14, also known as MKP6) in POCD produced by isoflurane in aged rats [37]. They showed that DUSP14 overexpression inhibited IL-1β, TNF-α, IL-6, and NLRP3 levels, as well as pyroptosis, and improved cognitive dysfunction in aged rats after isoflurane anesthesia, suggesting that DUSP14 may play a neuroprotective role in POCD by regulating NLRP3 inflammasome-mediated pyroptosis. In another study, Ma et al. showed that the knockdown of Su(var)3-9, enhancer-of-zeste, and trithorax domain-containing protein 7 improved cognitive impairment and ameliorated cell pyroptosis, which is associated with the inhibited release of inflammatory cytokines, and suppresses the activation of the NLRP3 inflammasome in the hippocampus in isoflurane-induced aged mice [46].

### 3.1.2 Sevoflurane-induced POCD

Shao et al. examined the neuroprotective effects of Chikusetsu saponin IVa (ChIV) against sevoflurane-induced neuroinflammation and cognitive impairment in old rats [94]. ChIV, a plant-derived molecule, has a wide range of pharmacological effects on the central nervous system, cardiovascular and cerebrovascular systems, immunological system, and the treatment and prevention of malignancies [95]. Behavioral and cognitive tests, such as the Morris water maze test, novel object recognition test, and Y-maze test, were used in this study. The results demonstrated that pretreatment with ChIV reduced sevoflurane-induced neurological dysfunction, apoptosis, and neuroinflammation. Additionally, sevoflurane administration raised the expression levels of NLRP3, ASC, caspase-1, IL-1β, and IL-18 proteins in the rat hippocampus.

Nevertheless, ChIV pretreatment removed this increment. This neuroprotective impact of ChIV is connected with its ability to disrupt the NLRP3/caspase-1 pathway, which was verified by in vivo experiments. Additionally, pretreatment with MCC950, a small-molecule medication that directly inhibits the NLRP3/caspase-1 pathway, augments the neuroprotective effects of ChIV [94].

### 3.1.3 Recurrent propofol-induced POCD

Propofol is a common anesthetic intermittently provided to elderly individuals for sedation during gastrointestinal endoscopy [19]. Older rats got repeated therapy with propofol once a day to imitate this clinical setting, which developed cognitive impairment. This study aims to evaluate the time-course effects of recurrent propofol anesthesia on cognitive performance in aged rats. The results demonstrated that propofol therapy daily, but not every nine days, led to cognitive impairment and neuronal death. Additionally, propofol administration boosted the activation of NF-κB and the NLRP3 inflammasome [96].

Additionally, daily propofol therapy elevated NF-κB p65 phosphorylation and upregulated IL-1β, IL-6, and TNF-α protein levels in the hippocampus and serum. Eventually, daily propofol administration elevated the NLRP3 and caspase-1 p20 levels in the rat hippocampus. Conversely, pretreatment with Bay-11-7082, a selective inhibitor of the NLRP3 inflammasome and an NF-κB inhibitor, decreased neuronal damage and cognitive impairment, which is related to NF-κB/NLRP3 inflammasome activity [96]. The results demonstrate that NLRP3 inflammasomes are implicated in recurrent propofol-induced POCD.

### 3.2. Function of the NLRP3 inflammasome in POCD produced by anesthesia and surgery

The investigations, as mentioned earlier, used animal models treated exclusively with anesthetics. Several laboratories have further studied the role of the NLRP3 inflammasome in POCD produced by a combination of anesthesia and surgery.

Jiang et al. examined the involvement of triggering receptors expressed on myeloid cells 2 (TREM2) and the accompanying NLRP3 inflammasome in POCD produced by anesthesia/surgery in mice [45]. TREM2 provides neuroprotective benefits against neuroinflammatory reactions [97]. TREM2 expression decreased after the induction of anesthesia/surgery, with increases in NLRP3 inflammasome activation and IL-1β expression and a decline in the neuronal survival markers PSD-95 and BDNF, suggesting that the NLRP3 inflammasome is involved in TREM2-mediated neuroprotection in the POCD animal model induced by anesthesia/surgery. Moreover, mitophagy antagonizes inflammasome activities [98]. Mitophagy is a specific type of autophagy that refers to a cellular process that selectively eliminates aged and damaged mitochondria via the specific sequestration and engulfment of mitochondria for subsequent lysosomal destruction [99]. Consistent with the rise in NLRP3 inflammasome activities, anesthesia/surgery reduces PINK1 and Parkin protein levels and key mitophagy regulators. Additionally, TREM2 overexpression attenuates POCD and enhances PSD-95 and BDNF expression, which were connected to mitophagy and reduced activities of the NLRP3 inflammasome and IL-1β.

Sun et al. evaluated the effect of electroacupuncture (EA) on POCD induced by partial hepatectomy in elderly mice [38], demonstrating that EA reduced IL-1β and IL-6 levels and blocked the activation of the NLRP3 inflammasome and NF-κB. Conversely, NLRP3 activation abrogated the effects of EA therapy on cognitive function [38].

Ye et al. evaluated the effect of honokiol (HNK), which has several organic protective actions associated with mitochondrial ROS (mtROS) and mitophagy in POCD produced by sevoflurane plus surgery [100]. The results demonstrated that HNK administration improved the protein levels of the autophagy and mitophagy markers, including LC3-II, Beclin-1, Parkin, and PINK1, after sevoflurane/surgery treatment. Additionally, HNK attenuated mitochondrial structural damage and lowered mtROS and MDA production, which are closely related to NLRP3 inflammasome activation [100]. Furthermore, HNK-mediated mitophagy reduced NLRP3 inflammasome and neuroinflammation activation in the hippocampus. The introduction of 3-MA, an autophagy inhibitor, eliminated the neuroprotective effects of HNK on mitophagy and NLRP3 inflammasome activation [100].

κ-opioid receptor (KOR) agonists have been proven to decrease inflammatory responses in several clinical situations [101, 102]. Most recently, the KOR agonist, U50488H, has also been indicated to be crucial in the therapy of medically generated neuroinflammatory responses [101, 103]. Song et al. studied its effects on POCD, neuroinflammation, and microglial polarization in rats exposed to cardiopulmonary bypass (CPB) [104]. CPB stimulates the NLRP3 inflammasome and elevated pro-caspase-1 production, which promotes pro-IL-1β and pro-IL-18 expression. The KOR agonist prevents hippocampus damage caused by CPB, inhibits NLRP3, and changes microglia from the M1 to the M2 state [104]. Consequently, the KOR agonist suppresses the inflammation mediated by microglia and improves POCD via the NLRP3/caspase-1 signaling pathway [104].

Zheng et al. explored the role of P2X7 receptor protein, which activities stimulate potassium efflux, leading to NLRP3 inflammasome assembly and pyroptotic cell death, in a mouse model with carotid artery exposure under anesthesia with isoflurane, sevoflurane, or desflurane [105]. They evaluated the varied effects of these anesthetics and discovered that desflurane anesthesia reduced neuroinflammation and cognitive impairment compared with isoflurane anesthesia after surgery. Additionally, the ionized calcium-binding adapter molecule 1 and IL-1β were raised in the hippocampus following surgery/anesthesia, yet, isoflurane was associated with a higher risk than desflurane. Additionally, surgery led to increased expression of P2X7 receptors and its downstream protein caspase-1, and P2X7 receptor inhibition alleviated neuroinflammation and cognitive impairment. Consequently, the P2X7 receptor likely mediated the postoperative neuroinflammation and cognitive impairment; however, how it altered inflammasomes was not explored.

## 4. Novel hypothesis of inflammasomes involvement in the surgical site and multiple organs in addition to the brain

**Figure 3.**
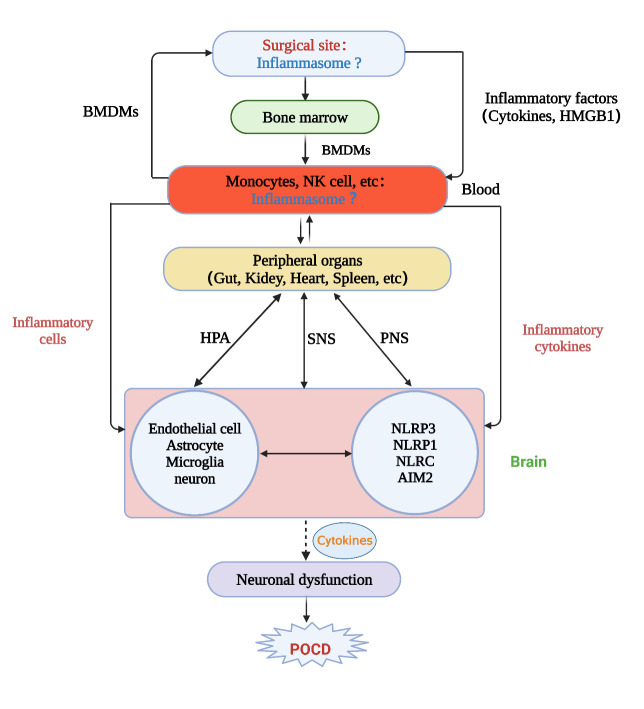
**Diagram presenting the novel hypothesis of POCD induced by surgery and anesthesia**. Tissue injuries in the peripheral organs result in the release of pro-inflammatory cytokines and cells, which cause BBB breakage and penetrate the brain parenchyma, leading to brain inflammation mediated by microglia and, finally, neuronal dysfunction and POCD. We hypothesize that inflammasomes are formed not only in the brain but also in the surgical site, circulating blood, and other peripheral organs. In addition, the novel hypothesis also proposes that multiple types of inflammasomes participate in POCD development.

After reviewing the overall structures and functions of various types of inflammasomes and the current evidence about the potential role of specific NLRP3 inflammasome in neuroinflammation induced by POCD, we now raise a novel hypothesis about POCD development (Fig. 3). This hypothesis explains how surgery can cause POCD via local site inflammation, blood circulation, systemic inflammation, breaching the blood-brain barrier (BBB), bidirectional effects between the brain and peripheral organs, multiple inflammasomes in the blood and brain, and neuronal dysfunction in the brain (Fig. 3).

First, major surgery can cause local tissue injury or damage, triggering a local inflammatory response. Recent studies have suggested that injuries to the skin and muscle tissues can result in local caspase-1 and IL-1β activities, indicating the involvement of inflammasomes [43, 106]. Tissue injury releases inflammatory factors, such as HMGB1 and other cytokines, into the circulation, stimulating bone marrow to release monocytes [107], which can be recruited by the injured tissue at the surgical site, further contributing to the inflammatory response. We speculate that local inflammation and potential inflammasome activities at the surgical site are among the primary causes of systemic inflammation, leading to neuroinflammation and POCD.

We further hypothesize that when local injury tissue releases inflammatory factors into circulation, it activates monocytes [108], neutrophils [108], NK cells [88], and platelets [109, 110], which may then form inflammasomes within these cells, leading to more systemic inflammation and release of bone marrow-derived monocytes (BMDMs). BMDMs and circulating inflammatory cytokines can access the brain via damaged BBB or areas lacking fully functional BBB, such as circumventricular organs [111], and penetrate brain tissue, leading to neuroinflammation. These cascades ultimately lead to oxidative stress, further exacerbating BBB damage, and synaptic dysfunction, contributing to POCD.

The central nervous system and the peripheral organs or tissues, including the immune system, are regulated in both directions, with inflammation impacting the brain via sensory neurons and the central nervous system (CNS) regulating peripheral immunity [112]. Surgery trauma often results in pain, an unpleasant sensory and emotional experience associated with actual or potential tissue damage. Pain is a complex physiological response that involves the activation of specific sensory neurons, called nociceptors, which are responsible for detecting noxious stimuli (such as mechanical, thermal, or chemical stimuli) and transmitting this information to the central nervous system [113].

When pain occurs due to surgery trauma, it triggers sensory neuronal activities that initiate a cascade of events [114]. Nociceptive information is first transmitted from the peripheral nervous system to the spinal cord via primary afferent neurons. These neurons synapse with second-order neurons in the dorsal horn of the spinal cord, which then relay the information to various regions of the brain, including the thalamus, somatosensory cortex, and limbic system.

Inflammatory reflexes are neural circuits consisting of afferent and efferent impulses in the vagus nerve that control the output of the innate immune system [115]. The lymphoid organs of the immune system are densely innervated by a peripheral neural network that modulates inflammatory responses by transmitting information to and from the central nervous system [116]. A typical neural reflex circuit is composed of sensory neurons that communicate information about peripheral changes to CNS interneurons and motor neurons that transmit efferent signals to peripheral tissues. Cognitive deficits may result from central nervous system inflammasome activity activating the HPA axis, the sympathetic nervous system, and the immune system, resulting in a cycle of brain inflammation and immune system abnormalities. In response to circulating inflammatory mediators, pattern recognition receptors or cytokine receptors expressed on these sensory neurons or the chemosensory cells in the accompanying vagal paraganglia activate vagal sensory neurons. Lastly, afferent vagus nerve fibers mediate the sensory arc of the inflammatory reflex, in which they detect disturbances in peripheral immunological homeostasis and relay the information to the CNS in a mediator-specific manner [117].

Surgical trauma not only causes inflammation at the site of the procedure but also often results in infection [118, 119]. Various DAMPs and PAMPs produced by injured cells and tissues can stimulate many types of inflammasomes, in addition to the NLRP3 inflammasome. In addition, the complex environment in the brain may also trigger activities of many types of inflammasomes. Therefore, it is necessary to investigate whether other types of inflammasomes are also involved in surgery-induced local and systemic inflammation.

Taken together, this theory posits that when inflammasomes are activated at the surgical site, local inflammation develops, which subsequently leads to systemic inflammation, a bidirectional immunological response, a breach of the BBB, and neuronal dysfunction in the brain, which activates inflammatory reflexes forming a vicious cycle between the brain and peripheral inflammation, culminating in POCD. This concept emphasizes the bidirectional contact between the CNS and peripheral organs and immune cells in the periphery. It indicates that inflammation in the periphery may result in immunological dysfunction and feedback on the brain, hence aggravating cognitive issues.

## 5. Potential research directions for POCD

**Figure 4.**
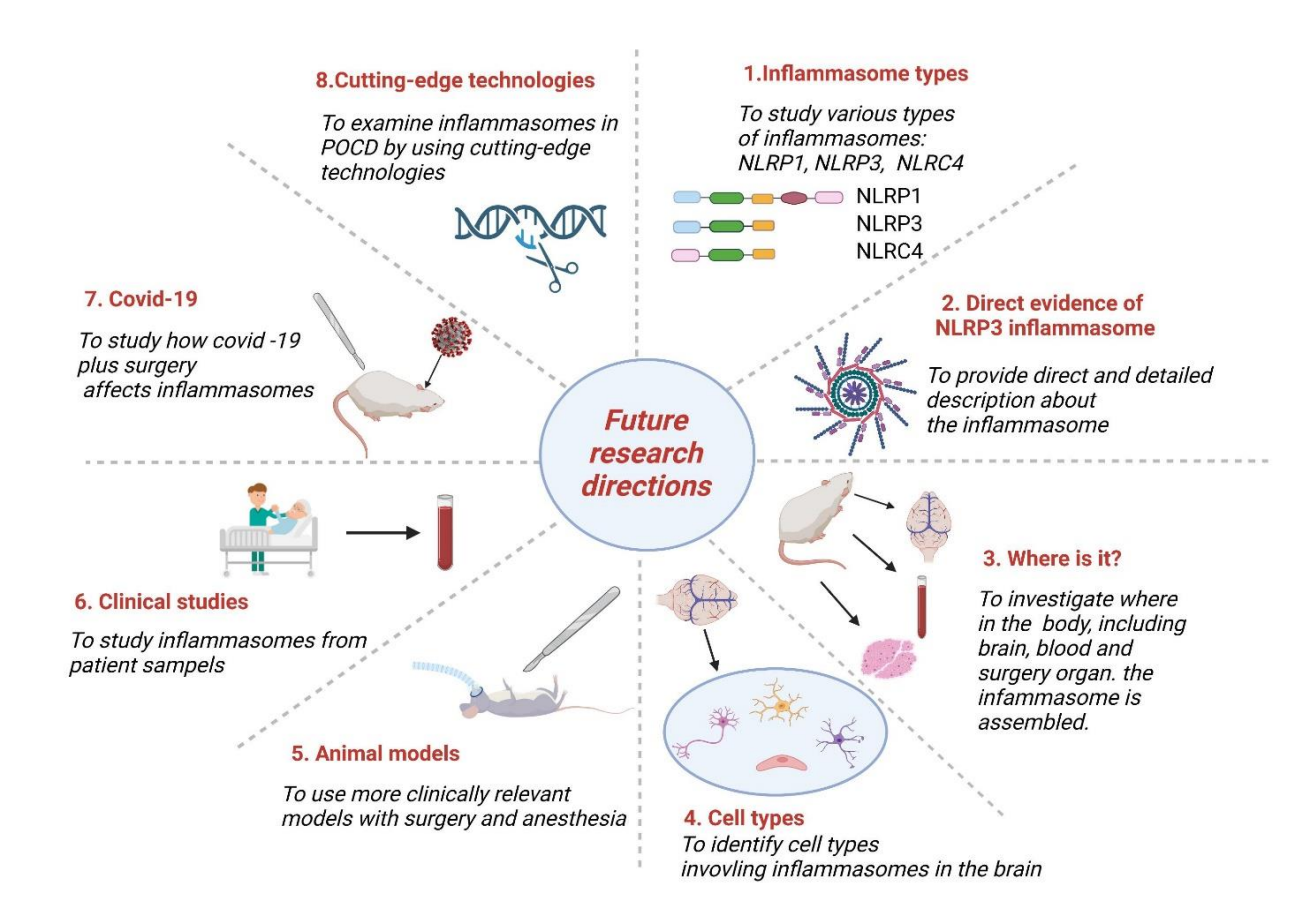
**Identification of future research directions for the role of inflammasomes in POCD**. The eight research areas identified for future research include inflammatory types, direct evidence of the NLRP3 inflammasome, the location of inflammasomes in the body, cell types associated with inflammasome activity, animal models, COVID-19 implications, clinical studies, and the application of advanced technologies.

Following the discussion on the novel hypothesis about POCD progression, we have identified several fields for future research that could further elucidate the underlying mechanisms and potential therapeutic targets (Fig. 4). Below, we elaborate on these areas and their implications for future research.

### 5.1. Explore various types of inflammasomes in POCD

Prior studies have mainly addressed the potential role of the NLRP3 inflammasome in POCD's pathophysiological mechanisms. Whether other types of inflammasomes are also implicated in POCD has yet to be studied. Among numerous inflammasomes, it appears that the activators for NLRP1, NLRP3, NLRC4, AIM2, and pyrin are those pathogenic elements derived from bacteria and viruses [120]. These inflammasomes may also be related to POCD because bacteria and viruses’ infections are often implicated after surgery.

Indeed, in addition to NLRP3, expressions of NLRP1 [121] and AIM2 [122] have also been found in neurons of the CNS. Although bacteria and viruses may not exist in the brain after major surgery under anesthesia, we cannot entirely discount the potential that other endogenous pathogenic pathways can stimulate their activity. Prior investigations have demonstrated that amyloid-β aggregates can activate NLRP1 [123, 124], which cleaves caspase-1 into its active components, leading the process of IL-1β and IL-18 into maturation. The same study also indicated that NLRP1-mediated caspase-1 activation produces pyroptosis [123, 124]. Moreover, several investigations have demonstrated that substantial AIM2 inflammasome activation was detected in neurodevelopment [67, 125], and abnormalities in AIM2 result in anxiety-related behaviors in mice [53, 126]. The AIM2 inflammasome also contributes to CNS homeostasis by regulating gasdermin-D activity [67]. Additionally, the role of the AIM2 inflammasome was examined in stroke-induced cognitive impairment in elderly mice [66], which demonstrated that AIM2 mRNA and protein, combined with caspase-1, IL-1β, and IL-18, were enhanced in the hippocampus and cortex after stroke. The cell types involving the AIM2 inflammasome include microglia and endothelial cells. Consequently, the authors concluded that the AIM2 inflammasome controls inflammation and pyroptosis after stroke [66].

Most recently, Sun et al. examined the activation of NLRP1, NLRP3, NLRC4, and AIM2 inflammasome in old POCD mice. They detected a considerable increase in NLRP3 and AIM2 mRNA levels but not in NLRP1 or NLRP4. Moreover, only the enhanced NLRP3 expression could be reduced by electroacupuncture therapy. This is further validated at the protein level by western blotting. These observations suggested that EA therapy can decrease the activation of NLRP3 inflammasome in old POCD mice [38]. Nevertheless, the role of AIM2 inflammasome in POCD requires further studies.

Since other types of inflammasomes can be detected and activated in different pathological brains, future studies should investigate whether these different inflammasomes, in addition to the NLRP3 inflammasome, contribute to the pathological mechanisms in POCD.

### 5.3. Further direct evidence and extensive descriptions of the impact of the NLRP3 inflammasome on POCD development are required.

As discussed, most previous studies have offered indirect and fragmented data on the NLRP3 inflammasome in POCD. Direct evidence on the NLRP3 inflammasome is sparse, and a complete description of the genesis of the NLRP3 inflammasome in POCD is inadequate. The NLRP3 inflammasome has three parts: the receptor or sensor NLR protein NLRP3, the adaptor ASC, and pro-caspase-1. The NLRP3 protein has three domains: the LRR domain, NACHT domain, and PYD. The amino-terminal PYD domain of the NLRP3 sensor facilitates homotypic associations with ASC, which through CARD-CARD contact further recruits pro-caspase-1 [41]. After the receptor or sensor hallmark activation and oligomerization, ASC assembles into a large protein complex called a "speck," a sign of inflammasome activation [127]. The ASC speck has a size of around one μm, which can be employed as a marker of inflammasome activation [128]. NLRP3 inflammasome activation leads to cleavage of pro-caspase-1 into active caspase-1, which then attracts pro-IL-1β and pro-IL-18 and transforms them into mature IL-1β and IL-18, which are released into the extracellular space.

Earlier investigations have revealed that IL-1β, IL-18, and active caspase-1 proteins are detectable in POCD [29, 92] and that caspase-1 inhibitors ameliorate POCD symptoms [37, 94], hinting that the NLRP3 inflammasome may be involved in POCD. However, these protein expressions are merely indirect evidence for the NLRP3 inflammasome because they can also be created by other types of inflammasomes [129]. Furthermore, IL-1β can be generated through inflammasome-independent pathways [130]; thus, the detection of IL-1β alone cannot be used as solid evidence for NLRP3 inflammasome activation. Furthermore, although relatively direct evidence suggested that caspase-1 inhibitors [29], NLRP3 knockdown, and NLRP3 inflammasome inhibitors attenuate POCD pathology [36], these results are scattered among different studies, and few studies have systematically and thoroughly investigated the existence of the NLRP3 inflammasome in POCD.

In the future, more focused research is needed to study the involvement of NLRP3 inflammasome in POCD. To evaluate inflammasomes in POCD, we must first determine the gene and protein expression of important inflammasome components, such as NLRP3, ASC, pro-caspase-1, pro-IL-1β, and pro-IL-18, as well as their cleaved counterparts, active caspase-1, IL-1β, and IL-18. This determination can be accomplished by PCR, western blotting, and ELISA [29, 92, 131]. Moreover, as inflammasome activation leads to pyroptotic cell death, cell death morphology can be examined using microscopy [132]. Moreover, as the ion outflow of K^+^ and Ca^2+^ drives inflammasome activation, fluctuation measurements at intracellular and extracellular levels may provide hints for inflammasome activity. Alternatively, to address the role of the NLRP3 inflammasome in POCD development, gene mutation of NLRP3 inflammasome elements can help. Additionally, directly targeting the NLRP3 inflammasome with small-molecule medicines to treat POCD can provide proof of its participation in POCD [89]. Finally, direct observation of the ASC speck, a hallmark of inflammasome development and activation, would give solid evidence of inflammasome participation in POCD.

### 5.4. Investigate inflammasomes in peripheral organs and the surgical site

Prior studies have studied that the NLRP3 inflammasome occurs in the brain. However, whether it also impacts the peripheral organs, especially the circulating blood and surgical sites, has not been explored in POCD. Because POCD is generated through systemic inflammation, the necessity of understanding inflammasome activity in the surgery site and circulation should be stressed. Furthermore, as surgical trauma occurring in peripheral organs and tissues results in ischemia/reperfusion or hypoxia to the organ or tissue itself [133], inflammatory responses arise in the surgical site [134]. Thus, inflammasomes also likely regulate tissue inflammation after surgery.

Additionally, the release of HMGB1 proteins, ATP, and ROS from the injury site into the circulation leads monocytes from the bone marrow to be discharged into the circulation [135], and these monocytes may be activated before accessing the brain. Activated circulating monocytes may release IL-1β and IL-18 via inflammasome activation, resulting in systemic inflammation. Consequently, we postulate that the NLRP3 inflammasome assembles in surgical organs, circulating blood, and the brain. Future studies should test this concept because if the NLRP3 inflammasome impacts the operative organ and circulating blood, therapeutics can be created to target inflammasome-associated cascades in these peripheral tissues or organs.

In addition to investigating inflammasomes in the brain, the surgical site, and blood, future research should evaluate the activation of inflammasomes in other peripheral organs. For example, surgical trauma leads to the release of DAMPs and PAMPs, which can activate inflammasomes in the brain and peripheral organs, such as the liver, lungs, and spleen. Activating inflammasomes in peripheral organs can lead to the generation of pro-inflammatory cytokines and other inflammatory mediators that can enter the circulation and contribute to systemic inflammation.

Furthermore, future studies should investigate the link between inflammasome activation at the surgical site and inflammasome activation in the brain. Activating inflammasomes at the surgical site can induce systemic inflammation that contributes to neuroinflammation and cognitive impairments in the brain. The systemic implications of inflammasome activation on the development of POCD could be better comprehended if the cross-talk between inflammasomes in peripheral organs and the brain were investigated.

Future research should investigate the activation of inflammasomes at the surgery site, in the circulating blood, and in peripheral organs in animal models and humans. This could provide new insights into the systemic effects of inflammasome activation on the development of POCD and identify new therapeutic targets for preventing and treating this condition.

### 5.5. Discover cell types containing inflammasomes in the brain

The cell types activated by the inflammasome in POCD remain unknown. Typically, inflammasomes form in monocytes, macrophages, microglia, and epithelial cells [133]. As microglial cells are the predominant resident immune cells in the brain, inflammasomes were presumed to form in microglia in most prior POCD investigations; however, in some POCD studies, the cell origins for inflammasome activation were not established. In addition, recent research has demonstrated that inflammasomes also regulate the functioning of other CNS resident cells, such as neurons [133], astrocytes [136], oligodendrocytes [137], and endothelial cells [90]. In POCD, however, it has yet to be determined if these cell types, in addition to microglia, participate in inflammasomes.

Several investigations have shown that neurons are the source of inflammasome activity in neurodegenerative disorders. For example, using Nlrp3^A350VneoR^ mice, a recent study found that dopamine neuron-specific activation of the NLRP3 inflammasome caused neuronal death [138]. Moreover, according to a second study [139], the absence of parkin activity results in the activation of the NLRP3 inflammasome and the death of dopamine neurons.

Astrocytes also contribute to inflammasome activation. In a study using male mice with a human mutant superoxide dismutase one variation as an animal model for ALS [140], astrocytes were identified as the predominant cell type expressing NLRP3 components. At the late phase of EAE, astrocytes exhibited AIM2 inflammasome activity [69].

Moreover, NLRP3 inflammasome is associated with vascular endothelial cell dysfunction and mortality [141]. Prior research has demonstrated that IL-1β, the primary product of inflammasomes, promotes the activation of secondary inflammatory mediators, such as IL-6 and C-reactive protein, and increases the release of adhesion molecules and chemokines in endothelial cells [142]. In endothelial cell death, NLRP3 inflammasome-mediated pyroptosis has also been identified [84]. In addition, inflammasome activation results in endothelial barrier failure, a crucial mechanism underpinning numerous inflammation-related illnesses [143].

In summary, in addition to microglia, recent investigations have demonstrated that neurons, astrocytes, and endothelial cells exhibit inflammasomes [136, 144, 145]. Microglia are not the only significant cell type for POCD pathogenesis [146, 147]; astrocytes and endothelial cells are also essential for POCD pathogenesis, and neuronal dysfunction is the last step in POCD. Given that the majority of CNS cell types contain the crucial components of inflammasomes, it is appropriate to investigate whether other cell types play essential roles for inflammasomes in mediating the development of POCDs.

### 5.6. Study more POCD animal models

POCD occurs following major surgery, which requires anesthesia; however, the independent contribution of anesthesia or surgery to the development of POCD is unknown.

Lai et al. evaluated the effect of anesthetic duration and surgical trauma on perioperative neurocognitive disorders (PND) and neuroinflammation [148]. According to the findings, surgical trauma, but not anesthetic, contributed to the development of PND and neuroinflammation. Nonetheless, because the unique effects of anesthesia and surgical trauma are frequently intertwined, it is essential to investigate how the combination of anesthetic and surgical trauma affects inflammasome activity in POCD [135]. Remarkably, some investigations on inflammasomes in POCD have been performed using animal models using only anesthetic. However, whether drugs alone can cause cognitive impairment is still debatable [149].

Many studies indicate that injecting anesthetic alone impairs spatial memory, causes neuroinflammation, and induces rodent death [150-155]. Yet, some other POCD animal investigations found no impact of the drug alone on cognitive impairments, including memory and learning function [92, 156-160]. Therefore, the activity of inflammasomes in various surgical procedures should be compared. A few investigations have studied the combined effects of anesthesia and surgery on POCD in mouse models. Included among the forms of surgery are partial hepatectomy [38], cardiopulmonary bypass (CPB) [104], and abdominal surgery [100].

Nonetheless, the effects of surgery on brain dysfunction are frequently separated into those associated with cardiac and noncardiac surgeries [161], as the effect of cardiac surgery on brain dysfunction is distinct from that of noncardiac surgery [162]. The cessation of blood flow to the brain as a result of cardiac surgery can directly cause brain damage. One study evaluated the effects of cardiac vs. abdominal surgery on POCD and neuroinflammation and found that their effects were distinct [149]. The study revealed that abdominal surgery primarily affects brain regions connected with the hippocampi, whereas heart surgery is related to changes in a wider range of brain regions [149]. Future research may combine anesthesia and surgery with POCD models that are more clinically useful. In addition, different noncardiac surgeries may also have varying impacts on cognitive performance, as the processes by which they affect brain function may vary depending on the surgically treated organ. Consequently, future research should examine differing effects between cardiac and noncardiac procedures and between different noncardiac surgeries.

### 5.6. The possibility for clinical research to confirm animal model findings

Systemic inflammation has been examined in blood samples collected from patients undergoing various surgical procedures while under anesthesia [163], but the existence of inflammasomes has not been addressed. Pooled cases of POCD (31%) occur after total hip arthroplasty (THA) [164], according to a meta-analysis, suggesting that POCD is frequent following THA. Patients with POCD having THA showed significantly greater inflammatory markers, including CRP, S-100B, IL-1β, IL-6, and TNF-α, than patients without POCD. Nevertheless, other meta-analyses indicated that POCD is related to IL-6 and S-100 but not IL-1β and TNF-α [165, 166]. Dexmedetomidine, a sedative, has dramatically reduced IL-6 and TNF levels in POCD patients [167]. Despite the significance of inflammation in POCD, few clinical studies have investigated inflammasome components in the blood of POCD patients. Among the analyzed inflammatory cytokines, IL-1β is the most related to inflammasomes; nevertheless, its role in POCD in clinical patients is contested, as some studies have not discovered it in POCD [45]. Regardless, IL-1β activity is always detected in animal models of POCD. There is a significant gap between animal and clinical research to comprehend the involvement of inflammasomes in POCD. Rather than focusing primarily on animal research, future studies should determine whether inflammasomes contribute to POCD in human patients.

Although animal models have offered essential insights into the role of inflammasomes in the pathophysiology of POCD, the applicability of these results to people remains to be discovered. Clinical research is required to confirm the results of animal models and create viable treatments for POCD.

Clinical investigations could provide helpful information regarding the occurrence and severity of POCD in humans and the association between inflammasome activity and cognitive impairment. Research might also examine the efficacy of therapies that target inflammasome activation, such as particular inhibitors of inflammasome activation, antioxidants, and BBB protectors.

Moreover, clinical research should examine the association between inflammasome activity and other risk factors for POCD, including age, comorbidities, and surgical procedures. The possibility exists that inflammasome activation may interact with other risk variables to raise the risk of POCD.

Surgical patients' blood or cerebrospinal fluid (CSF) could be analyzed for inflammasome-related biomarkers as a viable method for clinical trials. This could provide information regarding the activation of inflammasomes and the magnitude of the inflammatory response in response to surgical stress. In addition, clinical studies could potentially study the effects of therapies on the levels of these biomarkers and their connection with the onset of POCD.

In conclusion, clinical investigations are necessary to corroborate the results of animal models and create effective treatments for POCD. Furthermore, these investigations could provide helpful information regarding the occurrence and severity of POCD in humans and the link between inflammasome activation and cognitive dysfunction. In addition, the study of inflammasomes in clinical populations may result in the development of more tailored treatments for this ailment.

### 5.7. Potential implication of COVID-19 in POCD

The COVID-19 pandemic brought on by severe acute respiratory syndrome coronavirus 2 (SARS-CoV-2) has had and will continue to impact human life world significantly [168, 169]. The incidence of complications, such as acute respiratory distress syndrome, septic shock, and ischemic stroke, is more significant in infected patients undergoing emergency surgery [162]. These difficulties continue even if surgery is performed thirty days following a positive COVID-19 test [170]. In addition, patients undergoing surgery are more susceptible to infection with the COVID-19 virus because their immune systems are depressed due to surgical damage [171].

Research has revealed that individuals with COVID-19 infection have an increased risk of neurological consequences, such as delirium and cognitive dysfunction. The activation of inflammasomes in response to COVID-19 infection probably contributed to the emergence of these neurological problems. Moreover, patients who have recovered from COVID-19 infection may be at a higher risk of getting POCD after surgery. The long-term implications of COVID-19 disease on the immune system and the brain are not fully understood; nonetheless, COVID-19 infection may have lasting effects on inflammation and cognitive function, increasing the risk of POCD after surgery.

Although it is unknown if infected individuals undergoing surgery have a higher incidence of POCD complications, some researchers have hypothesized that viral infection increases the incidence of POCD complications among surgical patients [172]. Wei et al. hypothesized that SARS-CoV-2 disease combined with anesthesia/surgery might result in considerable neuroinflammation, mtROS upregulation, and amyloid-beta buildup, which can lead to hippocampus damage [173]. Moreover, they hypothesized that POCD might increase ACE-2 expression in the hippocampus, making it more vulnerable to COVID-19 virus infection, as ACE2 is the virus' entry point into the human body [174].

According to some studies, inflammasomes have been implicated in COVID-19 [175]. SARS-CoV-2 activates inflammasomes directly or indirectly, including cytokines such as IL-1β directed by inflammasomes, NLRP3 inflammasome activation, and observations of ASC specks and gasdermin-D amino-terminal processing and release [176]. Although inflammasomes are implicated in both POCD and COVID-19, COVID-19 infection may exacerbate the effects of surgery for patients who acquire POCD. Future research should study how COVID-19 influences the involvement of inflammasomes in POCD development.

Emerging research indicates that COVID-19 infection can result in the activation of inflammasomes and the release of pro-inflammatory cytokines, including IL-1β and IL-18. This may contribute to the formation of a hyper inflammatory state, also known as the "cytokine storm," which may lead to systemic inflammation and multi-organ dysfunction. In addition, infection with COVID-19 may further aggravate the activation of inflammasomes in response to surgical trauma, resulting in an increased risk of POCD.

Hence, it is essential to investigate how COVID-19 infection impacts inflammasome activation and its relationship and risk with surgery, as this could assist in identifying patients at elevated risk of POCD and developing tailored therapies to prevent and treat this condition. Future research should examine the occurrence and severity of POCD in patients with a history of COVID-19 infection, as well as the link between inflammasome activation, systemic inflammation, and cognitive dysfunction. In addition, the study of inflammasomes in patients with COVID-19 infection could lead to the development of new therapeutic options for this condition, such as using inflammasome inhibitors or other immunomodulatory treatments.

### 5.8. Use of cutting-edge technologies and methodologies to examine inflammasomes in POCD.

New chances to research inflammasomes in POCD have emerged due to methodological and technological developments. Possible technologies and methods are listed below:

1) Single-cell RNA sequencing: Single-cell RNA sequencing makes it possible to examine gene expression at the single-cell level and provides a deeper understanding of the role that different cell types play in POCD. The cell types that activate inflammasomes and produce inflammatory mediators in response to surgical stress may be discovered using this technique [68, 177].
2) To provide a more thorough knowledge of the role of inflammasomes in POCD, multi-omics analysis incorporates a variety of omics data, including genomics, transcriptomics, proteomics, and metabolomics [178]. This approach may be utilized to find new biomarkers for inflammasome activity and to comprehend the molecular mechanisms behind cognitive impairment brought on by inflammasome activity [179].
3) CRISPR/Cas9 gene editing: This technique allows for altering specific genes and gene products, making it possible to examine the role of inflammasomes in POCD with more focus. With this method, inflammasome-related genes in certain cell types may be selectively targeted or animal models created without key inflammasome components [180, 181].
4) Imaging techniques: Inflammasome activity in the brain and other organs may be monitored in real-time using imaging techniques such as positron emission tomography (PET) and magnetic resonance imaging (MRI) [122, 182]. For instance, MRI may identify changes in brain structure and function associated with inflammasome activity [182], whereas PET imaging can be utilized to visualize the expression of inflammasome-related biomarkers in the brain [183].

In summary, using contemporary tools and methods may open up fresh possibilities for inflammasome research in POCD. Furthermore, these tools and techniques might help us comprehend the role of inflammasomes in the pathophysiology of POCD and pave the way for creating ground-breaking therapies.

## 6. Conclusion and Summary

Researchers can develop new therapeutic targets and therapies for surgical patients with cognitive dysfunction if they understand how inflammasomes affect POCD. Hence, inflammasome research in POCD is crucial and has the potential to impact treatment management significantly. Future investigations into the role of inflammasomes in POCD have the potential to yield some benefits, including the identification of new therapeutic targets, advancements in diagnostic techniques, a better comprehension of the pathophysiology of POCD, the identification of fresh risk factors, and clinical application. Further research in this area is essential, considering the significance of inflammasomes in the pathophysiology of POCD. Modern instruments and methods may be used to study inflammasomes in peripheral organs and the surgical site. It may also be necessary to perform clinical research to validate the outcomes of animal studies and create POCD treatments that work. Patients with COVID-19 infections may be used in inflammatory research to develop new treatments for this condition, such as inflammasome inhibitors or other immunomodulatory medicines. Investigating inflammasomes in POCD has the potential to have a considerable impact on clinical practice, leading to the creation of cutting-edge diagnostic tools and specialized treatments that may enhance surgical patient outcomes. Hence, further study on inflammasomes in POCD is required, and researchers and medical professionals are urged to do so. This research may provide new insights into the underlying causes of cognitive deterioration after surgery and pave the path for the creation of more effective and targeted treatments. In the long run, it's anticipated that this research will improve patient outcomes and minimize the burden POCD places on patients and healthcare systems.
